# Interdisciplinary perspectives

**Published:** 2017

**Authors:** VL Purcarea

**Affiliations:** * "Carol Davila" University of Medicine and Pharmacy, Bucharest, Romania

From May 29 to May 31 2017, the imposing building of the Palace of Parliament hosted the fifth Edition of the Congress of “Carol Davila” University of Medicine and Pharmacy, Bucharest, the oldest and most important University of Medicine and Pharmacy in Romania, on the anniversary of 160 years since it was founded. 

Under the already famous motto, “Initiation, Evolution, Excellency”, 35 scientific sessions took place in five session rooms, in which prestigious speakers from Romania and from abroad presented their latest works and researches in the field of Medicine. The participation was numerous, as always, over 300 papers drawing the public’s attention through novelty and originality, the young researchers being remarked through enthusiasm and innovation. 

**Fig. 1:**
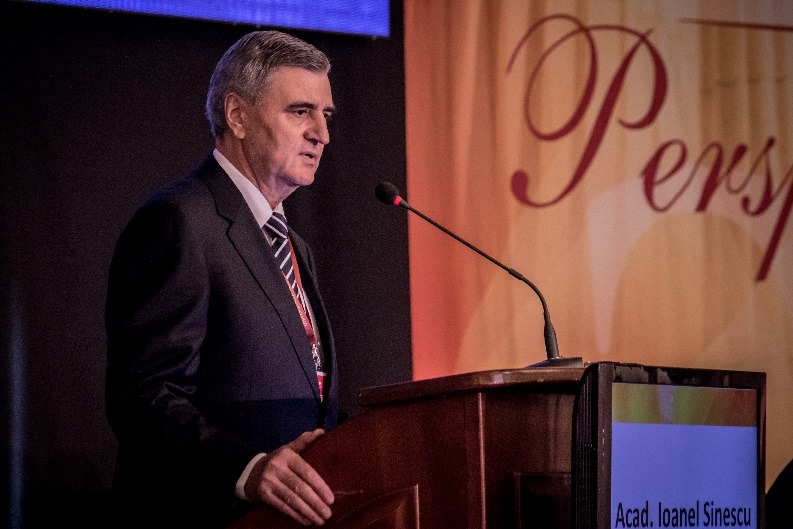
Opening ceremony of the Congress – Academician Ioanel Sinescu, Rector of “Carol Davila” University of Medicine and Pharmacy

The plenary sessions that should be mentioned are the following: Plenary session organized in collaboration with the Romanian Association of Urology: Updates in Urology; Multiple/ Plenary session Neurology: Alzheimer’s Disease – A multifactorial etiology disorder, A matter of complex interactions; Plenary session Cardiology: Heart failure in the comorbidities context; Plenary session Endocrinology: Parathyroid Diseases: Growing incidence or better diagnosis?; Plenary session organized in collaboration with the Romanian Society of Surgery and Traumatology: Research today and tomorrow. 

Moreover, the latest subjects that were discussed and that enjoyed a numerous participation and a special attention, are the following: Unrestricted Educational Grant from Biomedica: High-end molecular technologies in human diagnostic; Impact of atrial fibrillation in hematological, endocrine, gastroenterological, and neurological disorders; Interdisciplinary researches for innovative therapeutical systems development; From fundamental neuroscience to clinical practice in child and adolescent psychiatry; Patient-physician communication: Online communities, ethical perspectives, and patient satisfaction; Romanian Geriatrics and Gerontology at 65 years of presence in Europe: Prevention, intervention or anti-aging; Interdisciplinarity from the Dental Medicine perspective; Contemporaneity and interdisciplinarity in pediatric pathology; Multidisciplinary approach of the coronary artery disease: From the atherosclerotic plaque to management.

**Fig. 2:**
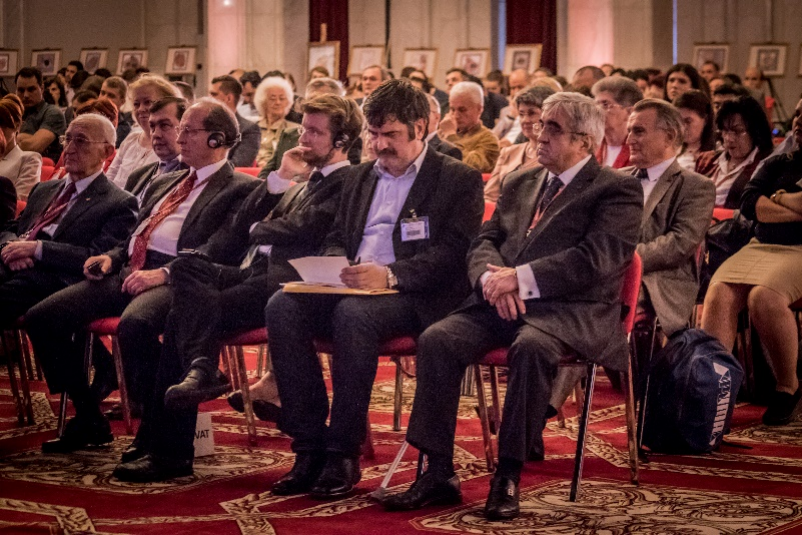
Participants in the Congress

Many prizes were awarded to the best of the best, such as Young Investigator Award (Preclinical Specialties), Young Investigator Award (Medical Specialties), and Young Investigator Award (Surgical Specialties), prizes that presupposed the financial support of their participation in international prestigious congresses. 

The opening ceremony was honored by some famous Romanian personalities such as Pavel Nastase – The Ministry of National Education, Serban-Constantin Valeca – The Ministry of Research and Innovation, Corina Pop –Secretary of State, Ministry of Health, Diana Paun – Presidential Consultant, Sorin Cimpeanu – President of the Rectors’ Council, Mircea Dumitru – Rector of University of Bucharest, Mihnea Costoiu – Rector of Polytechnic University in Bucharest.

A special moment of the opening ceremony was the one of unveiling the bust of the founder of the Romanian Medical School, General Doctor Carol Davila, which was sculpted by the famous sculptor, Rodion Gheorghita and given to “Carol Davila” University of Medicine and Pharmacy, Bucharest. 

**Fig. 3:**
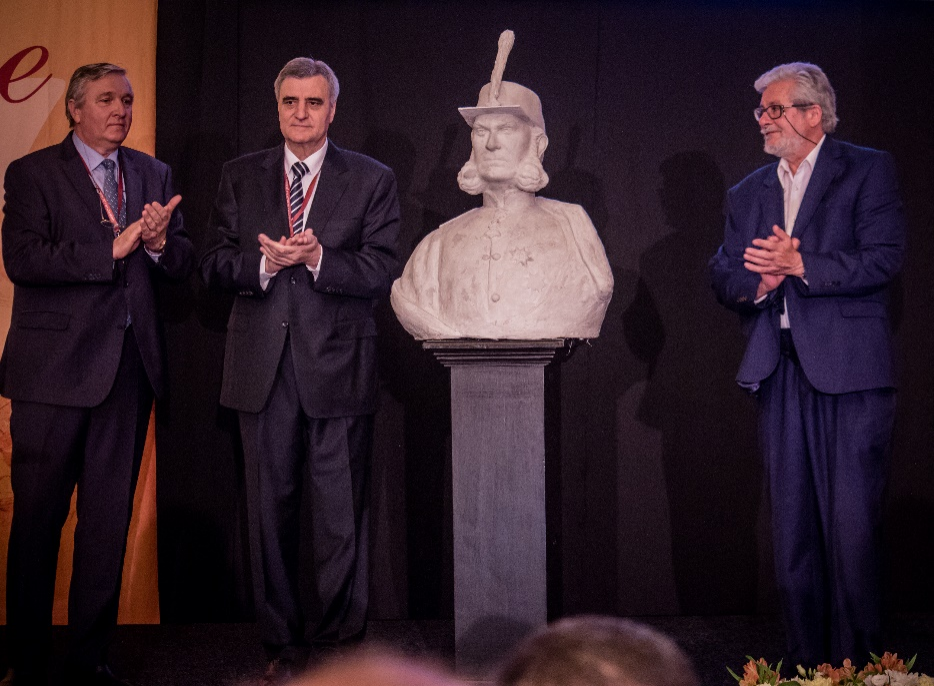
Unveiling the bust of General Doctor Carol Davila, the founder of the Romanian Medical School

In his speech on that occasion, the sculptor mentioned that among the 79 sculptures of the incomparable Constantin Brancusi, that no longer exist, that was the 26th, which he managed “to bring back to life” from a photo, his dream being the realizing of “an infinity column” of 50 meters, which he would place in front of the Palace of Parliament. 

The Congress also occasioned the awarding of the “Honoris Causa” title to some remarkable personalities in the field of universal medicine. 

**Fig. 4:**
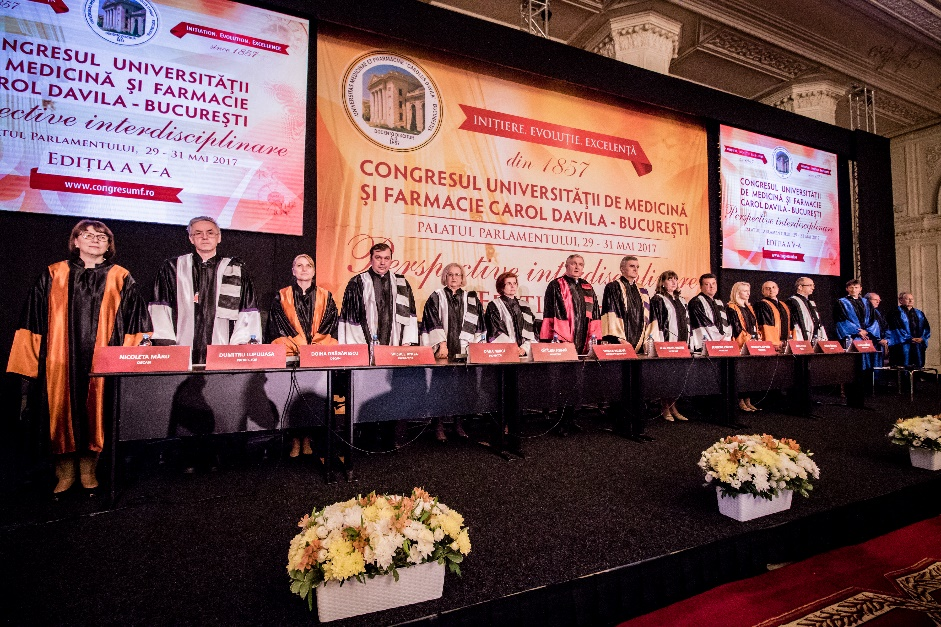
Members of the Senate of “Carol Davila” University of Medicine and Pharmacy in Bucharest, Romania

**ARNULF STENZL**

He was born on August 2, 1955 in Klagenfurt, Austria. 

From 1973 to 1980, he studied at the Medical School, Karl-Franzens University in Graz, and in 1980, he obtained the title of Doctor medicinae universalis (M.D.). 

From 1990 to 1992, he was Assistant professor in the Department of Urology, Inselspital, University of Bern, Switzerland, and from 1992 to 2002, he was Professor in the Department of Urology, University of Innsbruck, Austria. In 2002, he became Head of the Department of Urology, in Eberhard-Karls University Tuebingen, in Tubingen, Germany. 

He is member of many associations and committees in the medical field, such as Austrian Urological Association, German Urological Association, Austrian Association of Experimental Surgery, European Society of Urologic Oncology, and Endocrinology (ESUOE), Educational Committee of the Austrian Urological Association, Scientific Committee “Polish Journal of Urology”, Vice chairman of the MITT (Southwest German Industrial Research Corporation), since 2006 and Chairman of the Reinhard-Nagel Foundation Board, since 2013.

In his entire career in Medicine, he was offered many awards, such as AUA Annual Audio-Visual Award, in 1994 (San Francisco, USA); Honorary Member of the Bristol Urological Institute, Bristol, UK, in November 1997; Best study-center of the year 2005/ 2006, German Association of Urologic Oncology, in 2006; Werner-Stähler-memorial prize of the South-west German Association of Urology, in 2007, 2009, 2010 and 2011, etc. 

Moreover, he is the owner of some patents for important discoveries in the field of Urology, such as C-Trap, an implantable device that treats urinary incontinence. 

**Fig. 5:**
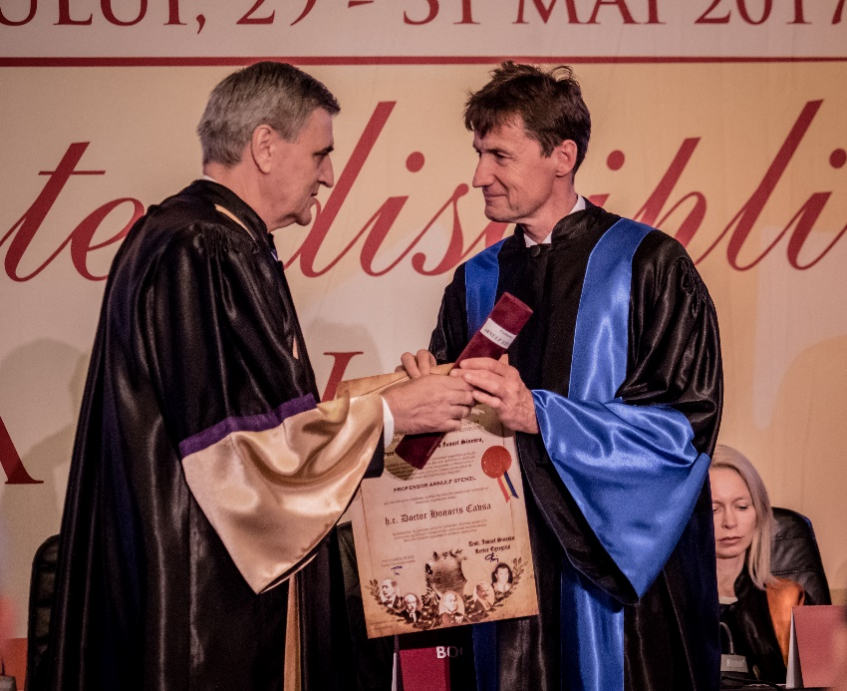
Arnulf Stenzl, M.D., PhD and Acad. Ioanel Sinescu

**PETAR M. SEFEROVIĆ**

He was born on January 20, 1951 in Belgrade. At present, he is the President-elect of the Heart Failure Association of the ESC, Corresponding member of the Serbian Academy of Sciences and Arts, Chair of internal medicine in Belgrade University School of Medicine and President of the Heart Failure Society of Serbia.

Regarding his education, he became Professor in 1996 in Belgrade University School of Medicine, in 1994 he became Head of Cardiology II, Department of Cardiology, University Institute for Cardiovascular Diseases, Belgrade, Serbian and Montenegro, and in 1994 he became Expert for Myocardial and Pericardial Diseases, Fed. Ministry of Science. 

Moreover, in 1982 he became Specialist in Internal Medicine. 

He is member of the European Society of Cardiology Activities such as HFA Board, Executive Committee of HFA Board, and was a Scientific Chairperson of the Heart Failure Association Annual Meeting in 2012. 

Even more, he is fellow of the European Society of Cardiology, member of the American College of Cardiology, honorary member of the Cardiac Promotion Society in Marburg, Germany, member of the Scientific Executive Committee of the International Academy of Cardiology and fellow of the Serbian Medical Association and Yugoslav Society of Cardiology.

As far as his research is concerned, he made studies regarding the heart muscle disease and pericardial disease, as well as invasive and interventional cardiology. 

**Fig. 6:**
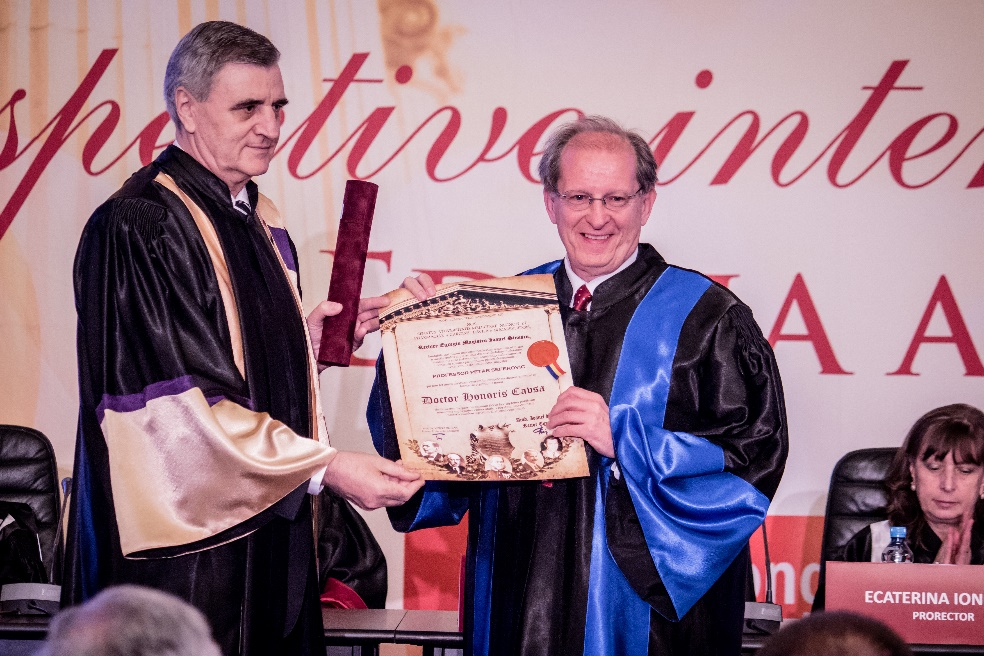
Petar M. Seferović, M.D., PhD and Acad. Ioanel Sinescu

**JENS RASSWEILER**

Prof. Rassweiler studied Medicine at the Universities of Stuttgart, Freiburg, and Tuebingen, in Germany. In 1982, he started his urological education at the Katharinenhospital Stuttgart and was directly involved in the clinical introduction of ESWL, URS, and PCNL. From 1988 to 1994, he served as Vice-chairman at the Medical School in Mannheim. He was the first German surgeon to perform a laparoscopic nephrectomy in 1992. In 1994, he became the Head of the Department of Urology at Klinikum Heilbronn, Academic Hospital of University of Heidelberg.

Prof. Rassweiler is an active member of various urological societies and member in the editorial board of a variety of international journals in the field of Medicine. In 1999, he received the Best Publication Award from the EAU. He also served in the board of the European School of Urology from 1999 to 2010. Since 2008, he has served as Chairman of the EAU Section of Uro-technology (ESUT). In 2009, he was President of the World Congress of Endourology & SWL, which he organized in Munich together with Prof. Christian Chaussy. He was President of the Engineering Society of Urologic Surgeons (EUS) and became President of the German Shock Wave Society in 2010. In the same year, he received the title “Doctor Honoris Causa” from “Victor Babes” University in Timisoara, Romania and, in 2013, he received the title “Doctor Honoris Causa” from “Iuliu Hatieganu” University of Medicine in Cluj-Napoca, Romania.

In 2016, he was elected Chairman of the EAU-section Office and recipient of the Gustav-Simon-Medal by the South-west German Society of Urology (SWDGU). In 2017, he was elected member of the American Association of Genitourinary Surgeons (AAGUS).

He is honorary member of the Endourological Societies of Hungary, Chile, Poland, Italy, and Turkey and honorary member of the Polish and Bulgarian Association of Urology. 

Professor Rassweiler has published more than 400 articles and more than 150 book chapters.

**Fig. 7:**
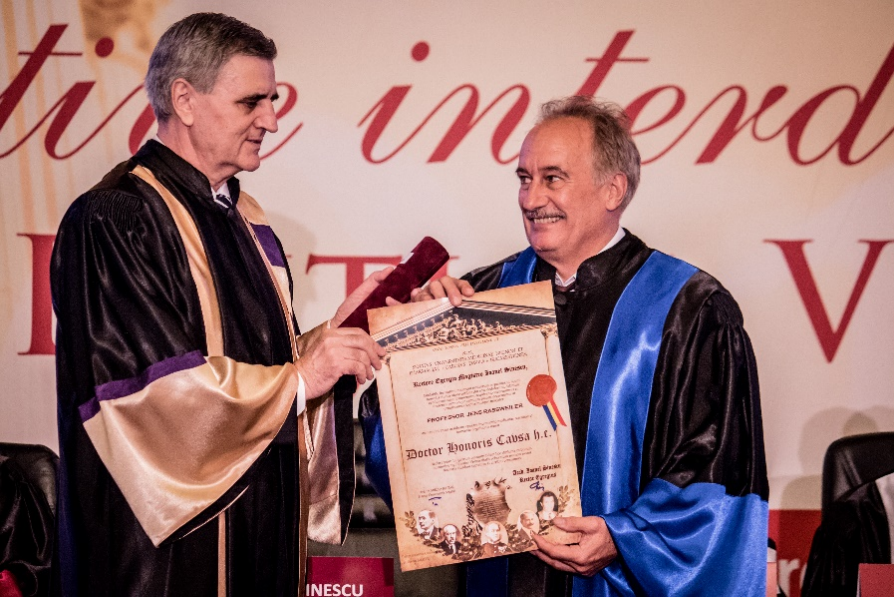
Prof. Jens Rassweiler, M.D., PhD and Acad. Ioanel Sinescu

**JOHN PAUL BILEZIKIAN**

He is currently Professor of Medicine and Professor of Pharmacology at the College of Physicians & Surgeons, Columbia University; Vice chair of the Department of Medicine for International Education and Research and Chief, Emeritus, of the Division of Endocrinology and Director of the Metabolic Bone Diseases Program at Columbia University Medical Center. 

In 1965, he graduated Magna Cum Laude from Harvard College, Cambridge, Massachusetts (B.A. in Biochemistry). In 1969, he became M.D., after graduating the College of Physicians and Surgeons, from Columbia University, in New York. 

He is member of the International Board of Medical Advisors, Yerevan State Medical University, Yerevan, Armenia; Director of the Annual Symposia on Osteoporosis, Yerevan State Medical University and American University of Armenia, Yerevan, Armenia. Moreover, from 2008 until present, he has been International Advisor of the Chinese Journal of Osteoporosis and Bone Mineral Research, and from 2011, he has been activating in the International Advisory Board of the Glucocorticoid Induced Osteoporosis Skeletal Endocrinology Group. 

From 2013 to 2015, he was member of the Scientific Statement Task Force of the Endocrine Society. 

Moreover, from 1983 to 1987 he was the Editor of “Endocrinology”. Starting from 2005 he has been Section Editor of the Journal of Women’s Health, member of the Editorial Board of “Journal of Clinical Densitometry” (from 2011), and, from 2016 he has been member of the editorial board of “Osteoporosis and Sarcopenia”. 

He was offered many awards and distinctions, among which the following should be mentioned: L.J. Henderson Prize in Biochemistry, by Harvard College, in 1965; Lifetime Achievement Award by the Armenian American Medical Society of California, in 2006; Laureate Distinguished Educator Award by The Endocrine Society, in 2014; Oscar Gluck Humanitarian Award, by the International Society for Clinical Densitometry, in 2014; Honorary Member, by the Russian Osteoporosis Association, in 2017, etc. 

He has an impressive number of citations in ISI Web of Science and an h-index of 83. 

**Fig. 8:**
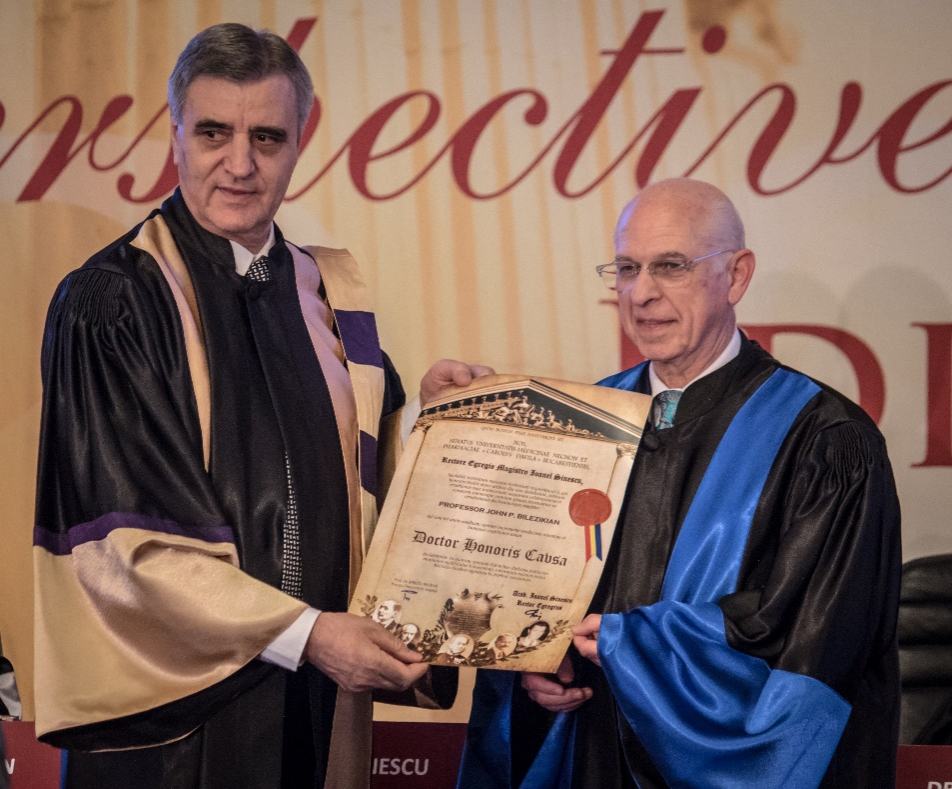
Prof. John Paul Bilezikian and Acad. Ioanel Sinescu

**ANDREAS LEITHNER**

He was born on April 23, 1974 in Vienna, Austria. 

From 1984 to 1998, he studied at the Medical Faculty of Vienna University. 

In 2005, he graduated venia docendi for “orthopaedics and orthopedic surgery” at the Medical University in Graz, thus obtaining the Austrian Specialist Diploma in Orthopaedic Surgery. From 2012 to 2016, he was Head of the Department of Orthopaedic Surgery, Medical University in Graz, and starting from 2017, he has been Head of the Department of Orthopaedics and Trauma Surgery, Medical University, in Graz. 

He is currently member of the Vienna Society of Physicians (since 2000); member of the German Society of Orthopaedic Surgery (DGOOC), (since 2009); member of the European Orthopaedic Research Society (EORS) (since 2012) and member of the Austrian Society of Orthopaedics and Traumatology (OGOuT), (since 2016). 

Moreover, he is General Secretary of the Austrian Society of Orthopaedic Surgery (OGO), since 2015, and Board Member of the same society. 

Since 2012, he has been the Editorial Board Member of “Sarcoma”; since 2015, he has been member of the “World Journal of Clinical Oncology” and, since the same year, he has been member of the “Scientific Reports”. Even more, he was the reviewer of the following journals: Acta Orthopaedica (2012-2016), American Journal of Clinical Oncology (2006-2012), NioMed Research International (2016), BMC Cancer (2012), etc. 

**Fig. 9:**
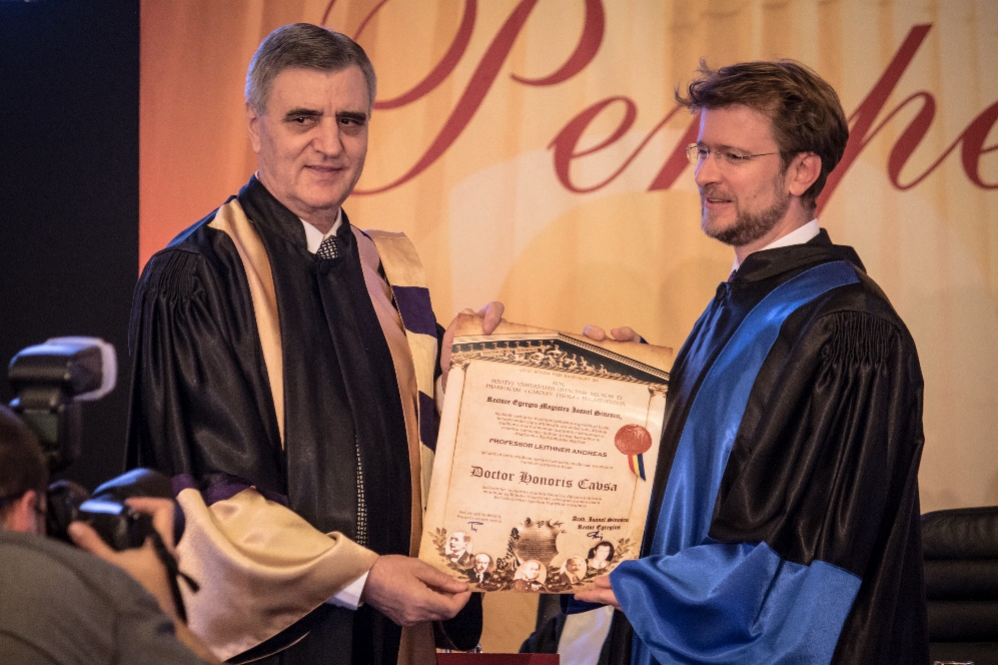
Andreas Leithner, M.D., PhD and Acad. Ioanel Sinescu

**BENGT WINBLAD**

Starting from 2000, he has been the Head of the Division for Neurogeriatrics, Department of Neurobiology, Care Sciences, and Society. Moreover, he has been the Director of the Clinical Trial Unit, Memory Clinic, Karolinska University Hospital, in Huddinge, starting from 2013, and Director of the Swedish Brain Power (Center for Early Diagnosis and Therapy Research for Neurodegenerative diseases – a Swedish Network), starting from 2005. 

From 2014 to 2015, he was Director of the Center for Alzheimer Research at Karolinska Institutet. Moreover, from 1987 to 2013 he was Chief Physician, at the Geriatric Clinic, Karolinska University Hospital, Huddinge, in Sweden. 

Regarding his professional activity, he is Chair of the EADC (European Alzheimer Disease Consortium), was Member of the Nobel Assembly for the Prize of Medicine and Physiology, in KI, in Stockholm (1988-2010) and also Chairman of the Department of Clinical Neuroscience and Family Medicine, in KI, Stockholm (1993-1999). 

Among all the awards he received, the following should be mentioned: 1997 – The Royal Swedish Academy of Medical Sciences Award for Dementia Research; 2002 – The Nordic Prize in Gerontology; 2008 – Award given by the Alzheimer’s Association, US, “Bengt Winblad Lifetime Achievement Awards in AD” to be given out every year at ICAD conferences; 2010 – Wajlit and Eric Forsgrens Award; 2013 – Research and Education Award 2013 by Queen Silvia Foundation; 2015 – The Swedish ”Hjärnfonden Jubilee Award”; 2016 – Alzheimer Association ”Henry Wisniewsky Life Time Achievement” award.

As far as his publishing activity is concerned, he published a total number of 1270 papers, he has a number of 53.591 citations, and his h-index is 114. 

**Fig. 10:**
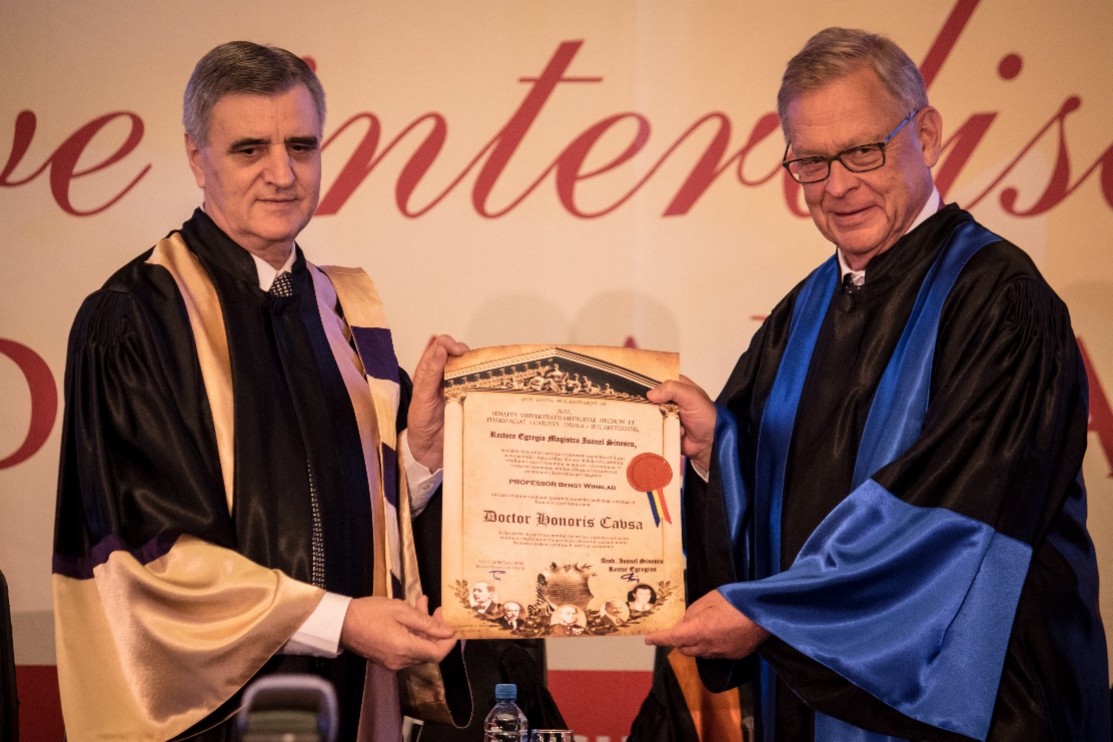
Bengt Winblad, M.D., PhD and Acad. Ioanel Sinescu

In the closing ceremony of the Congress, the Rector of “Carol Davila” University of Medicine and Pharmacy in Bucharest, Academician Ioanel Sinescu, especially thanked all the participants from the University, the whole country and abroad, and, at the same time, reminded them that “Carol Davila” University of Medicine and Pharmacy in Bucharest is the first university of medicine in Romania, which enjoys a well deserved prestige and recognition in the national and international academic environment, being a true leader of the Romanian medical education. 

**Fig. 11:**
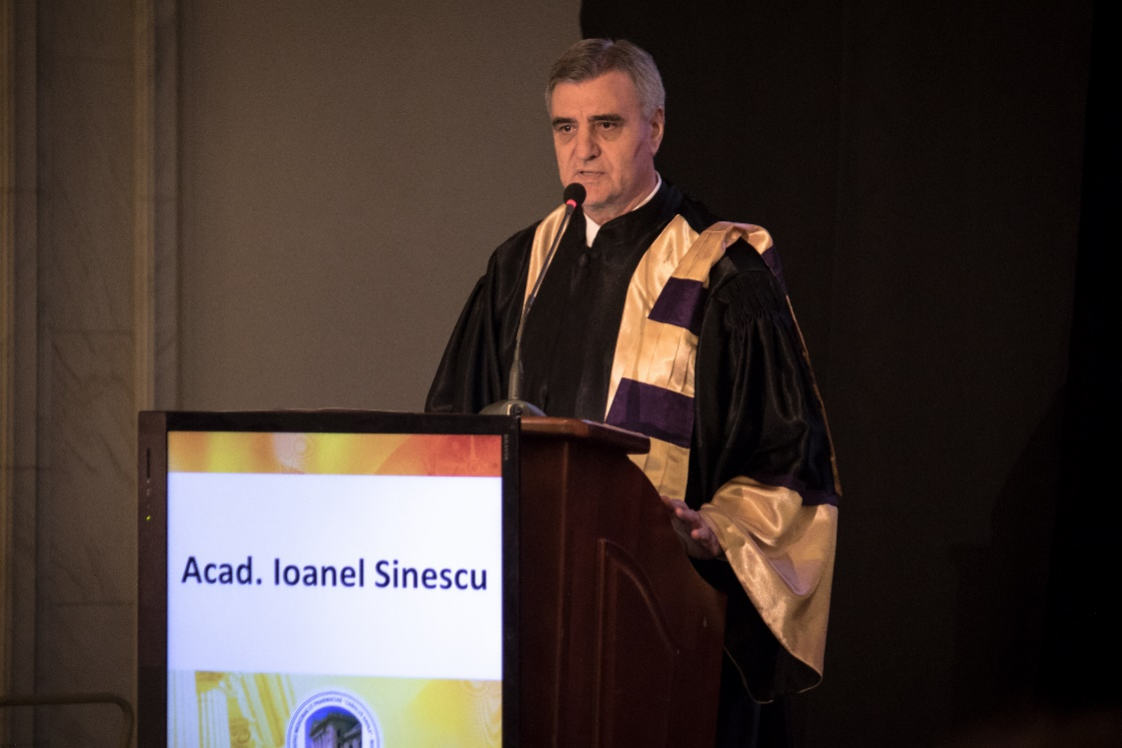
Closing ceremony of the Congress, Acad. Ioanel Sinescu, Rector of “Carol Davila” University of Medicine and Pharmacy

**Executive Editor** **Professor Eng. Victor Lorin Purcarea, PhD.**

